# RNA-ssisting immunity to heal the heart: a new frontier in therapeutics

**DOI:** 10.1097/CP9.0000000000000116

**Published:** 2025-06-24

**Authors:** Joel Gregory Rurik, Christian Le Phu, Julian Mustroph, Marcus Buggert

**Affiliations:** 1 Center for Infectious Medicine, Department of Medicine Huddinge, Karolinska Institutet, Stockholm 17177, Sweden.; 2 Department of Internal Medicine II (Cardiology), University Clinic Regensburg, Regensburg 93053, Germany.

**Keywords:** RNA, Immunotherapy, Immunology, Cardiovascular disease

## Abstract

Cardiovascular diseases remain the leading cause of death worldwide. Despite significant progress and the development of numerous effective drugs, substantial morbidity and mortality persist. This review highlights one potentially fruitful avenue for discovering novel therapeutics: leveraging ribonucleic acid (RNA) to tip the immunological balance toward tissue repair. Decades of research have primed the three disciplines of cardiology, immunology, and RNA drug development, to bring potent intersectional therapies to the clinic. We discuss both coding and non-coding RNA interventions across multiple cell types, such as monocytes, macrophages, and T cells, throughout different cardiovascular diseases. Altogether, advanced RNA-based medicines targeting the immune system are primed to transform how cardiovascular diseases are treated.

## INTRODUCTION

### Ribonucleic acid

**Figure 1.**
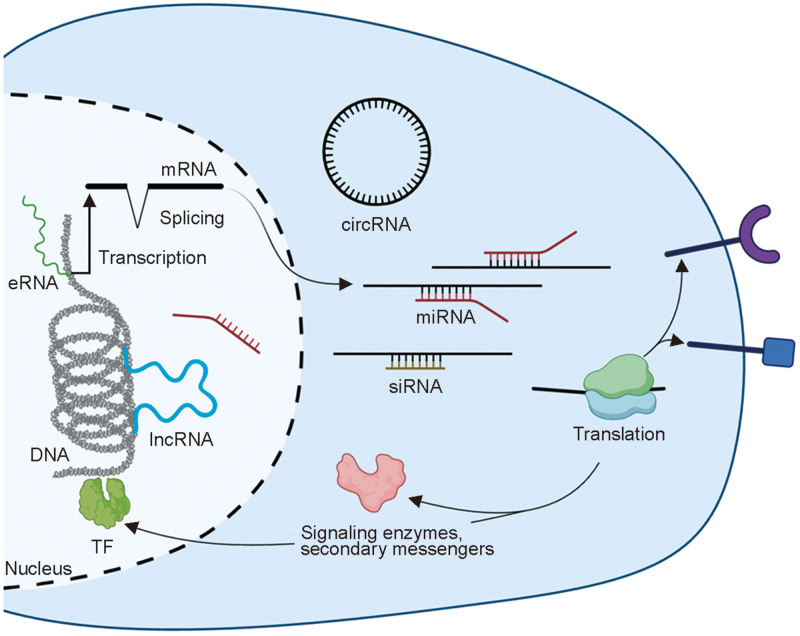
**Various RNA species regulate every level of the central dogma of biology.** LncRNA coordinate chromatin and can tether disparate genomic loci. eRNA assist in locus-specific transcription. mRNA is spliced by RNP complexes and exported from the nucleus for translation by the ribosome, which contains ribosomal RNA and utilizes transfer RNA to synthesize proteins. circRNA, miRNA, and siRNA can bind specific mRNA transcripts, causing their degradation. circRNA: circular RNA; DNA: deoxyribonucleic acid; eRNA: enhancer RNA; lncRNA: long non-coding RNA; mRNA: messenger RNA; miRNA: micro-RNA; RNA: ribonucleic acid; siRNA: small interfering RNA; TF: transcription factor.

The controlled passage of information is the fundamental core of life. Traditionally, the central dogma of biology is understood as deoxyribonucleic acid (DNA) being transcribed into a temporary messenger ribonucleic acid (mRNA), which is then translated into a functional and active protein. However, decades of research have revealed a far more complex and dynamic process. Every step, from DNA transcription to protein assembly, has multiple, redundant layers of regulation to refine and control the exact final suite of bioactive molecules (Figure 1). Importantly, every cell in the body at any given time leverages a unique set of control mechanisms to maintain its unique identity and enact its given function within the body, with many layers of control enacted by ribonucleic acid (RNA).

RNA molecules are powerful regulators that act throughout the cell^[1]^. In eukaryotes, RNA can be broadly classified as participating in translation and pre-/post-transcriptional regulation. In the nucleus, multiple species of non-coding RNA (e.g., enhancer RNA and long-noncoding [lncRNA]) enact epigenetic layers of control^[2]^, often by physically tethering desperate genomic loci or modulating RNA Polymerase II. These larger species of RNA help coordinate entire gene programs both by restricting and/or enhancing transcription. Nascent RNA from coding loci is then subject to splicing, which is a major hub of regulation, where long non-coding RNA are known regulatory players, especially to control key effector molecule production^[3]^. Furthermore, the introns that are spliced out of mature mRNA are often also potent biological molecules^[4]^. Finally, within the cytoplasm, transfer RNA and ribosomal RNA coordinate mRNA translation, while smaller RNA molecules (e.g., miRNA, small interfering [siRNA], and antisense RNA) can stabilize or degrade specific mRNA transcripts. An emerging theme in post-transcriptional control is that ribosomes display significant specificity for mRNA transcripts and how not all transcripts are equally translated^[5]^.

The incredibly complex, multilayered control systems endowed by evolution ensure that each cell state is tightly controlled. Because RNA are key players throughout the central dogma, they represent both potential targets and mechanistic opportunities for cardiovascular diseases.

The purpose of this review is to highlight potential RNA-based therapies across cardiovascular diseases. This broad concept involves either adding a therapeutic RNA molecule to or modulating endogenous RNA species/programs within a targeted cell type. These drugs include nucleic acid medicines like mRNA, lncRNA, siRNA, antisense oligonucleotides (ASO), and locked nucleic acids^[1]^ and any number of other chemicals on RNA. Often, there is direct overlap with therapeutic RNA targeting endogenous RNA, for example, siRNA silencing an injury-reactive, pro-inflammatory mRNA. To further contain the scope of this review, we have chosen to focus on therapeutics aimed at modulating the immune system and its multipotent effects across cardiovascular diseases. Furthermore, current RNA delivery vehicles, such as lipid nanoparticles (LNPs) and lipoplexes, are incapable of robustly transducing cells beyond the endothelial barrier (e.g., cardiomyocytes, smooth muscle, mural cells, or fibroblasts). Yet LNPs are capable of robust delivery to lymphocytes, hepatocytes, and endothelial cells among others^[6–9]^. Most importantly, we strongly believe that numerous important therapeutics are about to emerge from the rapidly expanding field of immunocardiology.

### Immunological participation in cardiovascular disease

**Figure 2.**
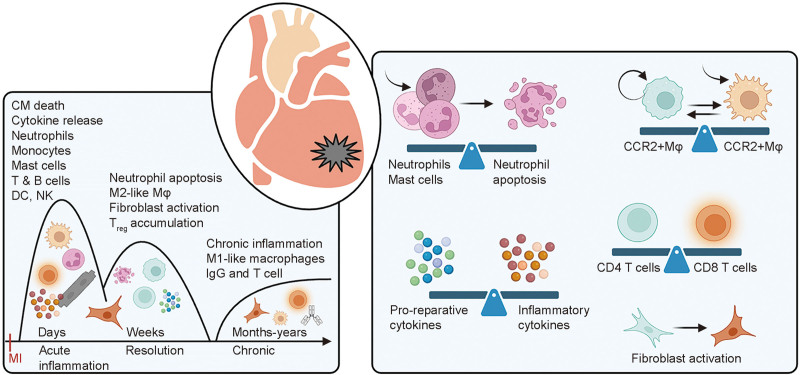
**Immune cell involvement following ischemic cardiac injury**. Ischemic injury causes widespread cardiomyocyte death and release of numerous inflammatory cytokines, chemokines, DAMPs, and PAMPs. In the early phases of injury response, the heart is flooded with neutrophils, mast cells, NK cells, monocytes, DCs, T cells, and B cells. Fibroblast activation peaks near the end of the first week and continues, alongside apoptosis of neutrophils and other granulocytes. Ideally, the myocardium then shifts toward a reparative milieu with many CD4 T_regs_ and M2-like polarized macrophages. The multifaceted immune responses each exist as a balance between inflammation and repair, which shifts over time. CCR2: C-C chemokine receptor 2; CM: cardiomyocyte; DAMPs: damage-associated molecular pattern; DC: dendritic cell; Mφ: macrophage; MI: myocardial infarction; NK: natural killer; PAMPs: pathogen-associated molecular pattern; Treg: regulatory T cell.

The immune system is an enormously complex, multifaceted network of cells and molecules designed to protect the body from foreign pathogens. In parallel, robust tolerance mechanisms are required to ensure that the potent killing activity of the immune system does not turn against healthy host cells, tissues, or commensurate microbiome constituents. A simplified interpretation of the immune system is that it is responding to deviations from homeostasis. Most frequently, this means responding to pathogenic infections, however, since all tissues and cells in the body are intimately connected, it is only logical that immune components respond to non-infectious tissue distress, including ischemic cardiac injuries. Indeed, most researchers and clinicians appreciate the inseparable participation of the immune system in various cardiovascular injuries and diseases (Figure 2)^[10–12]^. Furthermore, emerging experimental evidence at the intersection of immunology and cardiology calls into questions whether a certain cell type should be categorized as part of the immune system, the cardiovascular architecture, or both. Activated cardiac fibroblasts are a prime example, as they seem to serve as critical signaling hub(s) for both the adaptive and innate immune systems in addition to their canonical roles as extracellular matrix factories^[13–14]^. Furthermore, vascular lesion formation is driven by inflammation and hallmarked by pathogenic leukocyte contributions^[15–16]^. These will be further discussed in the following paragraph, but they serve as critical examples of how an integrated view of cardiac disease pathology will continue to yield innovative and impactful therapeutic approaches.

Our understanding of how the many immune cell types, cytokines, and chemokines participate in the damaged heart has advanced significantly in the past decades, yielding valuable insights and novel therapeutics^[11–12]^. A distinction should be drawn between non-ischemic diseases and ischemic injuries where the presence of dead cell debris, inflammatory signals (e.g., damage- and pathogen-associated molecular pattern [DAMPs and PAMPs]), and metabolite accumulation can act to inflame the tissue and recruit/maintain damaging cells. These damaging signals typically exacerbate tissue injury and inhibit myocardial recovery. Macrophages have been intensely studied in this context. While there is significant heterogeneity between macrophages, tissue-resident (C-C chemokine receptor 2 negative [CCR2−]) macrophages are self-sustaining population that generally promote tissue recovery^[17–18]^. In contrast, infiltrating monocytes differentiate into more damaging (CCR2+) macrophage subsets^[19]^. Similarly, multiple classes of T cells infiltrate the heart and can be cytotoxic, inflammatory, and damaging^[20–21]^, or regulatory and driving tissue recovery^[22]^. Cytotoxic T cells are rapidly recruited to the heart in myocardial infarction (MI) perhaps even in the immediate minutes after reperfusion, at least adhering to damaged endothelial cells^[23]^. In the days following MI, additional T cells accumulate in the myocardium where they can both propagate inflammatory damage or stimulate recovery^[11]^. Experiments in rodents suggest that antigen-specific T cells can inadvertently be trained to recognize and target heart epitopes, leading to ongoing damage^[20,24]^. B cells can also produce cardiac-specific antibodies which accumulate in the heart. The contributions of adaptive—anti-heart—T and B cells are not yet well understood in the context of human myocardial diseases^[25]^. The valvular damage in rheumatic fever is one of the better worked-out human cardiac diseases caused by an adaptive immune response (primarily a synergy between cross-reactive CD4 T cells and B cell-produced IgG arising from molecular mimicry)^[26–27]^. CD8 T cells have also been proposed to participate in ongoing injury after ischemic cardiac diseases, however, human-specific research is limited^[24,28]^, likely due to the lack of defined epitopes and longitudinal access to cardiac tissue. Interestingly, one of the proposed sarcomere proteins, Myh6, targeted by anti-heart T cells is not expressed by thymus endothelium^[29]^, suggesting that a pool of circulating precursors exist that are held in-check by peripheral tolerance, which may be overcome by significant myocardial damage, viral infection, or immune checkpoint inhibitor therapy to drive myocarditis and other myocardial pathologies^[28]^.

However, it should not be forgotten that the tolerance mechanisms built into the immune system are also active participants in cardiovascular disease (CVD) and are known to restrain damage and promote tissue recovery. Accordingly, regulatory T cells (Treg) also infiltrate the damaged heart and stimulate recovery^[22,30]^. Many other pro-reparative immune cells are actively recruited following injury^[11]^. Tissue-resident CCR2− macrophages have been particularly well studied for their role in promoting myocardial recovery. The balance between each pro- and anti-inflammatory subsets of cells is critical and nuanced, with nonspecific, broad-spectrum therapies that target the immunologic aspects of CVD showing limited or null results in clinical trials^[10–11]^.

Across non-ischemic cardiomyopathies (NICM) where cardiomyocyte death may be more limited, the contributions of the immune system are less well understood. These diseases include hypertrophic, dilated, and restrictive cardiomyopathies, alongside many other etiologies, affecting millions of people around the world. These diseases often show signs of chronic inflammation, although the precise pathologic mechanisms remain undetermined^[31]^. Disorders involving the extracellular matrix, such as amyloidosis, sarcoidosis, and especially fibrosis, which is prevalent across most cardiac diseases, appear to have immune components, especially hallmarked by low-level chronic inflammation^[20–21,32]^. Fibrosis and inflammation may also contribute to certain arrhythmia disorders. Furthermore, heart failure (HF), in its many diverse forms, has pathologic contributions from a chronically inflammatory milieu^[33]^.

Moving beyond the heart into the vascular tree, the diverse contributions of the immune system to vascular diseases have been well documented^[15,34]^. Plaque formation in atherosclerosis is driven largely by inflammation of the endothelium and its interactions with various inflammatory immune cells^[16]^. Plaques display heterogenous composition, with macrophages being especially prevalent. This inflammatory disease can also be driven by clonal hematopoiesis of indeterminant potential (CHIP) which can also initiate hematologic cancers^[35]^.

As the intertwined realms of CVD and immunology are being elucidated, one should not discount the contribution of metabolism to both systems. Atherosclerosis is a prime example of the interdependent mechanisms: excess low-density lipoprotein (LDL) drives vascular inflammation which kicks off the disease process involving vascular constituents and cells recruited from circulation^[34]^. Within the injured heart, during the reperfusion injury phase after ischemia, potent alterations in metabolite usage and electron transport chain utilization are thought to drive myocardial damage^[36–37]^. Furthermore, immune cells utilize different metabolic pathways given different niches or disease contexts^[38]^. Targeted RNA interventions may intervene in any of these pathways with potentially profound effects on CVD.

### Making RNA therapeutics a reality: decades of progress clear a formidable hurdle

The 2023 Nobel Prize in Medicine or Physiology was awarded to Dr. Karikó and Dr. Weissman for discovering that therapeutic mRNA synthesized with modified uridine avoids triggering an innate inflammatory rejection within transduced cells. The insight was made by in vitro synthesizing RNA with various modified bases found in native tRNA species. Incorporation of pseudomethyluridine was found to completely abrogate innate immune rejection within transduced cells^[39]^. This critical advancement has enabled safe and efficacious RNA therapies across all applications, from vaccines to gene editing therapies. Behind the Nobel limelight, hundreds of pioneers significantly contributed to the development of RNA therapies, including critical in vitro synthesis processes and purification methods^[40]^. For example, many chemically distinct nucleoside analogs can be incorporated into the synthesized RNA to significantly alter the cytotoxicity, synthesis and translation efficiency, and/or fidelity^[41]^.

The critical challenge for RNA-based pharmacotherapy is RNA gaining access into the appropriate target cell(s). To enable this, many advanced delivery vehicles have been formulated, and development is continuing at a rapid pace^[40]^.

**Figure 3.**
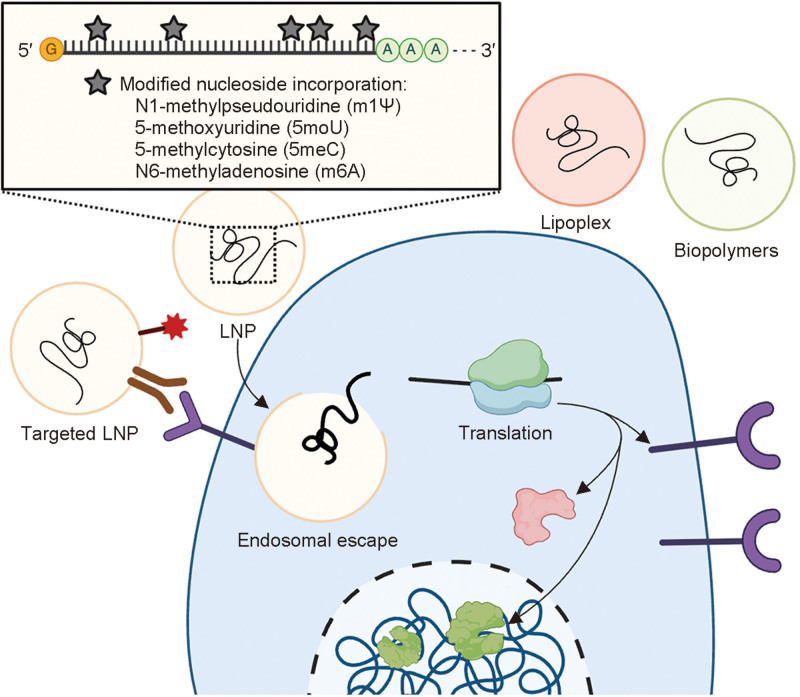
**RNA base modification and efficient delivery vehicles.** mRNA is often synthesized with modified bases such as methylpseudouridine, 5-methyoxyuridine, 5-methylcytosine, and N6-methyladenosine, among others to avoid activating innate sensors. RNA may also be synthesized with fluorescent dyes such as Cy5. Delivery of RNA to target cells typically requires efficient delivery vehicles. Numerous approaches have been developed, including LNPs, lipoplexes, and bio-compatible polymers. Delivery vehicles can be actively targeted to a specific cell type by incorporating antibodies, antibody fragments, ligands, nanobodies, etc. RNA is released to the cytoplasm by endosomal escape for translation by host machinery. A: adenine, representing the poly(A) tail; G: guanine, representing the 5’ cap; LNP: lipid nanoparticle; RNA: ribonucleic acid.

Lipid-based nanoparticles and lipoplexes are related vehicles for delivering RNA (Figure 3)^[42]^. Both have been used extensively in vaccines (e.g., COVID-19^[40,43]^), United States Food and Drug Administration (FDA)-approved siRNA therapies (e.g., Patisiran^[44]^), numerous clinical trials (e.g., PCSK9 gene disruption^[45]^), and proof-of-concept studies (e.g., in vivo generated anti-fibrotic chimeric antigen receptor [CAR]-T cells^[6]^). Cell and tissue targets of these vehicles can be altered by changing the lipid structure, composition, stoichiometry, or by adding active targeting motifs to the exterior. In addition to changing tissue delivery, these modifications can substantially alter the pharmacodynamics, breakdown, and clearance. Perhaps most importantly, each vehicle and ligand targeting chemistry has profound effect on the immune system. On one end of the adjuvant spectrum, MC3-containing LNPs utilized in the COVID-19 vaccines induce strong cytokine release and immune cell activation^[46]^. Although useful to induce viral protection, this adjuvant activity is not desirable in a CVD patient. Fortunately, chemical alterations to the vehicle can substantially mitigate the drug immunogenicity, while maintaining capacity for RNA delivery^[47]^. Current vehicles often include polyethylene glycol (PEG) which a subset of potential patients may develop antibodies against^[48]^. Therefore, the chemical parameters must be carefully assessed and fine-tuned for safety for the treatment of CVDs. Lipid-based vehicles have demonstrated RNA delivery to many cell types, including the liver, lungs^[49]^, spleen, endothelium^[9,50]^, cardiomyocytes after MI^[51]^, and many different types of lymphocytes, including hematopoietic stem cells (HSCs)^[8]^, T cells^[6–7]^, natural killer (NK), monocytes^[52]^, and macrophages^[52]^. As the challenges in RNA delivery are being solved and safely translated into the clinic, both non-coding and coding RNA therapies will likely enter the mainstream research channels en masse to tackle a broad swath of CVDs.

## NON-CODING RNA INTERVENTIONS IN CVDs

### Nucleus

Across development, health, and disease, many lncRNA coordinate gene expression^[53–54]^. There are tens of thousands of lncRNA representing a large fraction (>80%) of the transcriptome and remaining vastly under-studied. LncRNA exert regulatory control of transcription in numerous formats, many of which are involved in cardiovascular development^[55]^, homeostasis, aging, and disease^[54,56–57]^. The important conceptual theme across nuclear non-coding RNA are that they enact or fine-tune entire gene programs, often through chromatin/epigenetic interactions^[58]^. This makes them attractive targets for intervention. The therapeutic potential of lncRNA in CVD is largely underexplored, especially in the context of immune system. However, tantalizing insights into endogenous lncRNA responses to myocardial injuries have been reported and well-reviewed elsewhere^[53,59]^. For example, the hypertrophic response to non-ischemic injuries appears to be partially coordinated within cardiomyocytes by lncRNA-mediated chromatin alterations involving *MyHeart*^[60]^ and *Sweetheart*^[61]^. This suggests that an antisense RNA could be devised to disrupt these lncRNA, potentially diminishing cardiac hypertrophy. Throughout cardiac development and neural crest cell migration multiple lncRNA (*Upperhand* and *Handsdown*) are known to regulate expression of critical transcription factors like *Hand2*^[62]^ and influence 3D chromatin organization^[53]^. These lncRNA may be leveraged toward the goal of cardiomyocyte regeneration since each lncRNA helps coordinate gene regulatory programs, above and beyond modulating a single factor.

Several reports have identified similar lncRNA-centric mechanisms that control the phenotype of immune cell response to myocardial injury^[63]^. Monocytes are quickly recruited and known to differentiate into either inflammatory or reparative macrophages. Influencing the ratio between these broadly defined phenotypes dramatically aids the recovering heart. An inventory of lncRNA expressed in various macrophage populations from humans with cardiometabolic diseases found several thousand intergenic lncRNA, where many correlate with disease and activation status^[64]^. Additional studies have identified a role for *PVT1*, a lncRNA expressed in bone marrow-derived macrophages found in the heart after sepsis-induced myocarditis injury, capable of pushing these cells toward an inflammatory phenotype with knockdown proof-of-concept showing potential therapeutic applicability^[65–66]^. Furthermore, there is a connection between *PVT1* and several microRNA, which will be discussed in the following paragraph. Similarly, lncRNA *NEAT1*, is induced in human peripheral blood mononuclear cells (PBMCs) after MI, with mouse knockout studies suggesting a direct role in coordinating monocyte differentiation and cardiac recruitment^[67]^. Synthetic RNA-based therapies could be designed to interrupt these endogenous lncRNA, potentially discouraging lymphocytes from contributing to cardiac pathology.

LncRNA are also involved in vascular disease pathogenesis. For example, *RAPIA* has been identified as a potent positive regulator of pathogenesis in atherosclerotic lesion, and in macrophages, it supports inflammatory gene synthesis and overall cell survival^[68]^. Interestingly, like *PVT1*, one regulatory role of *RAPIA* is as a molecular sponge of miRNA-183-5p, alongside promoting the expression of genes of the integrin β1 pathway^[68]^. Another lncRNA known as *MAARS* is dramatically upregulated in pathogenic macrophages as they infiltrate aortic intima and strongly reinforces the inflammatory and anti-apoptotic pathways^[69]^. These lncRNA involved in atherosclerosis progression may be prime targets for therapeutic disruption with synthetic RNA-based drugs.

These discoveries, alongside significantly more examples^[63]^, provide evidence for potentially beneficial cardiovascular therapeutics based on lncRNA delivered to or disrupted within monocytes. Comparable lncRNA biology inevitably controls the inflammatory versus regulatory response in T cells following myocardial injury^[70]^. It is yet to be determined exactly which therapeutic lncRNA, or combination of non-coding RNA, needs to be delivered at what timepoint after a given injury to push lymphocyte phenotypes toward pro-regenerative. One role for lncRNA and certain circular RNA^[71]^ is to act as a sponge, removing specific miRNA from the cell.

miRNA are an enormous class of small non-coding RNA, typically 21-23 nucleotides, that are complementary to specific sequences in mRNA. miRNA are often highly specific to a given cell type and niche context. miRNA primarily operate in the cytoplasm (discussed later), however, many are preferentially located in the nucleus^[72]^. miRNA are thought to be capable of influencing large gene networks^[73]^. This facet of miRNA biology has largely been under-explored in the context of immunocardiology, however, early translational progress has been made in targeted interference to cardiomyocyte and cardiac fibroblast miRNA to stimulate tissue recovery after MI, electrical conduction correction^[74]^, and in HF^[75]^. In fact, an oligonucleotide-based inhibitor that blocks miR-132 is currently under clinical evaluation in patients with heart failure with reduced ejection fraction (HFrEF). miR-132 inhibitor was well tolerated in patients with the highest dose, reducing circulating levels of N-terminal prohormone of brain natriuretic peptide (NT-proBNP), suggesting efficacy and reinforcing the need for larger phase II-III trials (NCT04045405)^[76]^. In pre-clinical mouse models, over-expression of miR-132 alone within cardiomyocytes is capable of inducing HF phenotype and targeted blockade reversing HF symptoms in mouse and porcine models^[77]^. Within fibroblasts, miR-21 is thought to drive fibrosis and resulting cardiac hypertrophy through alterations to the extracellular signal-regulated and mitogen-activated protein (ERK-MAPK) kinase signaling, with silencing experiments suggesting miR-21 as a viable anti-fibrotic therapeutic target^[78]^. These are only two examples of many other miRNA that coordinate both homeostasis and disease recovery within the non-immune compartment of the heart^[59,75]^.

Interestingly, miR-21 is also involved in macrophages’ pathogenic response to hypertensive cardiac injury, where it was found to push cardiac macrophages toward an M1-like inflammatory phenotype, which were then capable of activating fibroblasts to further drive pathology^[79]^. Likewise, many miRNA such as miR-155^[80]^ and miR-125b have also been proposed to promote pro-inflammatory phenotype in macrophages^[81]^. Synthetic miRNA sponges, designed to remove endogenous miRNA^[82]^ by acting as a decoy, could be a therapeutic option to limit the conversion of macrophages to the M1-like phenotype in heart disease. On the other side, miR-146a, miR-24, miR30b, and many others negatively regulate responses to inflammatory signaling and promote a more regenerative, M2-like phenotype in macrophages^[81,83]^. Outside of macrophage and monocytes, miRNA also exert phenotypic control in cytotoxic T cells. In the realm of vascular diseases, miR-181a were downregulated in hypertensive kidney macrophages and endothelial cells, seemingly acting in conjunction with renin^[84]^. Studies in miR-181a knock-out mice suggest that the miRNA is pathologically important in both salt handling and blood pressure control^[84]^. With advances in RNA delivery vehicles, it may be possible to resupply miR-181a in this common disease as a nucleotide-based blood pressure regulator. As immunocardiology-focused RNA therapies are conceived and developed, caution is warranted due to the pleiotropic effects that many RNA have depending on the disease and cell type context^[85]^.

Thinking even more ambitiously within the nucleus, synthetic RNA molecules could be designed to alter the epigenome at specific genome loci. Potentially inspired by enhancer (eRNA)^[86]^ or lncRNA tethers^[87]^, totally artificial, RNA-based epigenetic regulators could be used to establish or enforce expression of certain genes or even genetic programs. For example, custom eRNA-like molecules could be designed to release paused RNA polymerase II to reinforce transcription of specific genes^[88]^, potentially master transcription factors controlling Treg phenotypes (forkhead box P3, FoxP3) or pro-reparative macrophages (signal transducer and activator of transcription, STAT6, krüppel-like factor, KLF4, etc.). As the challenges of synthesizing and safely delivering large RNA molecules to specific cells are being worked out, these types of innovative therapies will become feasible.

### Cytoplasmic, non-coding RNA

Within the cytoplasm, synthetic RNA molecules may be powerful therapeutic options by controlling translation. Inspired by natural lncRNA containing SINE elements (SINEUPs), artificial SINEUPs recruit heavy polysomes to specific mRNA, increasing protein concentrations anywhere between 2- and 5-fold, without altering the quantity of mRNA or global translation^[89–91]^. Comparable to the nuclear non-coding interventions described earlier, the SINEUP approach or similar could be developed to force protein accumulation of certain transcription factors, signaling molecules, or chemokine receptors to enforce pro-reparative phenotypes in, or cardiac recruitment of, specific immune cells. Importantly, this method complies with current cytoplasmic delivery limitations of lipid-based vehicles and avoids any genomic alterations which always carry the risk of genotoxicity^[92]^, malignant transformation, or unwanted germline modifications. Another therapeutic potential is to alter host ribosomal rRNA or tRNA to favor translation of target mRNA, given that ribosomal translation is non-random and an important layer of translational regulation^[5]^.

In addition, strong therapeutic potential exists to intervene on mRNA degradation pathways. As discussed previously, endogenous miRNA in animals often bind dozens of target mRNA^[93]^. In the cytoplasm this binding results in degradation or blocking translation of the mRNA. Often miRNA bind multiple mRNA within the same phenotypic network, posing a fascinating intervention strategy provided the correct miRNA can be identified. Dozens of miRNA have been proposed to control cell states across the immune system, only a few examples will be highlighted here.

One clear therapeutic opportunity is delivering miRNA to monocyte-derived macrophages to influencing their phenotype following cardiac recruitment. Several endogenous miRNA, including miR-139-3p^[94]^, and miR-182^[95]^, and others^[96]^ have been identified originating from extracellular vesicles that seem to promote pro-recovery phenotypes in macrophages. In each study, markers for M2-like macrophages were observed following miRNA activity leading to cardiac recovery. Outside of heart-specific studies, miR-24^[97]^, miR-145^[98]^, and many others^[99]^ have been identified as capable of influencing macrophage phenotype. It should be noted that many miRNA are context- and species-specific and therefore must be exhaustively tested in appropriate CVD models. Macrophages are perhaps the best-studied leukocytes in cardiac recovery; however, other responding cells likely contain comparable biology and therefore therapeutic potential. For example, miRNA could be modulated to influence T cell cytotoxicity versus regulatory phenotypes^[100–101]^, which is known to promote myocardial recovery^[30]^. Taken together, the combination of advanced synthetic delivery vehicles with engineered miRNA represents attractive cardiovascular therapies, with significant future therapeutic development potential.

miRNA sponges are designed to eliminate specific miRNA and can be delivered in various formats, including lentiviral and linear or circular RNA-based vectors^[102]^, to deplete certain miRNA within the cytoplasm, leaving the endogenous mRNA to be transcribed. Multiple target miRNA (miR-27a^[103]^, miR-155^[104]^, etc.^[96]^) that are known to drive pro-inflammatory phenotypes within macrophages^[99]^ and other immune cells could be depleted with this strategy to lower the drivers of inflammation. It is worth noting that some miRNA sponge approaches have been proposed for cardiac injury models^[71,105]^, but do not rely on immune-based mechanisms.

siRNA molecules are highly specific degraders of target mRNA. Multiple siRNA therapies have been approved by the FDA, first for transthyretin (TTR)-mediated amyloidosis (patisiran and vutrisiran) and then for liver metabolic disorders (givosiran and lumasiran)^[44,106]^. ASO are single-stranded versions of siRNA that also degrade a target mRNA. ASO can be further modified with tissue-homing ligands and be administered without a lipid delivery vehicle. For example, eplontersen is FDA-approved to treat hereditary TTR amyloidosis^[107]^. Properly timed siRNA-based therapeutics could precisely tip the balance of inflammation through a significant number of critical molecules across many cell types. One prime example is that blocking interferon regulatory factor 5 (IRF5) in macrophages reduces their inflammatory (M1-like) markers and supports cardiac recovery after MI^[108]^. Pro-inflammatory cytokines, chemokines, and cytotoxic molecules could be blocked with an siRNA approach, although knocking down any one molecule within the incredible complexity of immunocardiology may have limited efficacy. Furthermore, perhaps pathogenic lymphocyte recruitment into the heart could be limited by blocking adhesion molecule production within endothelial cells by leveraging targeted LNPs^[9]^. One could also consider targeted knockdown within the emerging brain-heart axis, which appears to influence myocardial recovery at least in part through polarizing monocytes^[109]^. Separately, blocking homing receptors within key subsets of damaging lymphocytes, such as CCR2+ monocytes or CD8+ T cells, may sufficiently skew the intramyocardial balance of inflammatory lymphocytes to promote tissue recovery. One attractive feature of siRNA in these applications is the temporary nature of inhibition, which could be timed to stimulate recovery or limit damage following acute myocardial injury without permanently abrogating an infectious disease response.

Aptamers are small nucleotide molecules, typically 20 to 100 base pairs, engineered to bind to and modulate target proteins comparably to monoclonal antibodies. Numerous aptamers have been proposed as biosensors, diagnostic tests, drug delivery vehicles, and therapeutics^[110]^, with only a few examples potentially impacting cardioimmunology highlighted here. Tissue necrosis factor α (TNF-α) was one of the first inflammatory cytokines identified in the pathogenesis of heart disease, yet an unspecific biological inhibitor (etanercept) failed to improve heart function in patients^[111]^. Aptamers with improved specificity for the inflammatory TNF-α pathway are in development^[112]^. Additional aptamers have been developed to interfere with key inflammatory cytokines such as interferon-γ (IFN-γ), interleukin (IL)-17A, IL-32, IL-2, and chemokines such as monocyte chemoattractant protein-1 (MCP-1, also known as chemokine ligand 2, CCL2), stromal cell-derived factor 1 (SDF-1, also known as C-X-C motif chemokine 12, CXCL12), and IFN-γ-induced protein (IP-10, also known as CXCL10)^[113–114]^. Furthermore, aptamers have been developed against toll-like receptors (TLR) including one that blocks TLR4, protecting the brain from inflammation following ischemic stroke (NCT04734548)^[115]^. Without stabilization, aptamers are quickly cleared from the body, making them good imaging probe candidates, for example, visualizing tenascin-C to identify atherosclerotic lesions^[116]^. Taken together, aptamers may prove therapeutically powerful agents to modulate or monitor inflammatory responses in heart disease.

### Coding (mRNA) interventions

**Figure 4.**
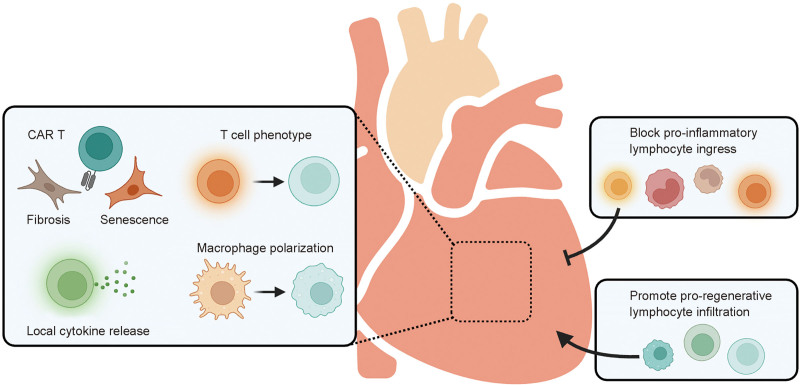
**RNA-based therapeutic strategies to tip the immunologic balance toward repair.** Pushing T cell and macrophage phenotypes toward pro-regenerative state is known to improve cardiac recovery. Naturally recruited leukocytes could be engineered to locally secrete anti-inflammatory cytokines. Anti-fibrotic and anti-senescent CAR T cells aid the heart by specifically eliminating pathogenic cells. Approaches to block infiltration of damaging, pro-inflammatory cells or boosting the recruitment and retention of pro-reparative cells can also strongly influence myocardial recovery. CAR: chimeric antigen receptor; RNA: ribonucleic acid.

Comparable to the seemingly infinite number of non-coding interventions introduced earlier, significant promise exists leveraging coding mRNA interventions across the immune system to promote cardiovascular recovery (Figure 4). Similar themes will be highlighted here, with therapeutic proteins introduced to key cells for modulating the immune involvement after cardiovascular injury.

As previously discussed, the proper balance of macrophage subsets is integral to regulating the immune response during cardiac inflammation, injury, remodeling, and recovery^[18–19,117–120]^. Cardiac macrophages are broadly classified into two subsets: CCR2− and CCR2+^[18,117,120]^. Tissue-resident CCR2− macrophages exhibit anti-inflammatory properties, proliferate locally, and play essential roles in vascular development and myocardial regeneration^[18,117,120]^. Conversely, CCR2+ macrophages primarily arise from infiltrating monocytes which are associated with pro-inflammatory activity, contributing to myocardial damage^[18,117,120]^.

This functional diversity is further exemplified by the classical sorting of macrophages into pro-inflammatory “M1-like” and anti-inflammatory “M2-like” phenotypes, although this dichotomy oversimplifies their complex roles^[117,121–122]^. M1-like macrophages are classically activated by pro-inflammatory stimuli, including TLR ligands such as lipopolysaccharides, cytokines like IFN-γ and TNF-α, and growth factors such as granulocyte-macrophage colony-stimulating factor (GM-CSF) or M-CSF^[121,123]^. These cells secrete nitric oxide, reactive oxygen species (ROS), and pro-inflammatory cytokines (e.g., IL-1β, TNF, IL-12), while also producing matrix metalloproteinases (MMPs)^[117,124]^. These inflammatory molecules are pleiotropic with effects throughout the myocardium, including interfering with critical cardiomyocyte electrophysiology and calcium handling^[125–126]^. M1-like macrophages exhibit phagocytic and antigen presentation activities which are critical in infectious disease responses and tumor suppression by promoting inflammatory responses^[117,121–122]^. In contrast, M2-like macrophages are activated by various anti-inflammatory factors, including IL-4 and IL-13^[121]^. They secrete anti-inflammatory cytokines such as IL-10 and CCL1, enhance extracellular matrix production, and stimulate angiogenesis and cell proliferation, thereby facilitating tissue repair and regeneration^[117,121]^. Importantly, macrophage polarization is a dynamic process, characterized by inherent plasticity that enables macrophages to transition between phenotypes in response to inflammatory signals within their microenvironment^[117–120]^. In the context of the CCR2 classification mentioned above, CCR2+ macrophages are predominantly associated with polarization toward the pro-inflammatory M1-like phenotype, whereas CCR2− macrophages are more closely aligned with M2-like anti-inflammatory functions^[11,117]^. For a more comprehensive understanding of cardiac macrophage subtypes and their activities, refer to the reviews by Ma *et al*.^[118]^, Kim *et al*.^[119]^, and Zuo *et al*.^[117]^.

Shifting macrophage polarization holds significant therapeutic potential across various pathological conditions^[117,119–120,127–128]^. For instance, in cancer therapy, promoting a shift from the M2-like to the M1-like phenotype is highly desirable. This transition enhances the pro-inflammatory response and collective anti-tumor activity^[52]^. Conversely, in cardiovascular pathologies such as MI or HF, encouraging M2-like polarization is advantageous given their central roles in reducing inflammation, promoting tissue repair, and mitigating cardiac damage in both acute and chronic conditions^[117,119]^.

One promising strategy to achieve macrophage polarization shifts involves utilizing mRNA delivery techniques to express specific factors that drive macrophages toward the desired phenotype^[52]^. This approach allows for precise modulation of macrophage behavior by transiently introducing mRNA-encoding cytokines, receptors, transcription factors, or other molecules that influence polarization^[52,121,129]^.

For example, Zhang *et al.*^[52]^ introduced a novel approach utilizing mRNA-based nanoparticles encoding the transcription factor IRF5 and inhibitor of nuclear factor kappa-B kinase subunit beta (IKKβ), which drive M1-like polarization^[52,130]^. This strategy was applied in mouse models of ovarian cancer, glioma, and systemic metastases using mannose-coated nanoparticles, which preferentially targeted CD206+ cells, particularly macrophages and monocytes^[52]^. Treated mice exhibited significant disease regression, highlighting the approach’s potential as an effective cancer therapy^[52]^. The converse approach should be developed to promote pro-reparative phenotype in cardiac diseases by encoding transcription factors which promote the M2-phenotype like STAT6, c-MYC, KLF4, peroxisome proliferator-activated receptor gamma (PPARγ), or GATA-binding protein 3 (GATA3)^[96,130–131]^.

Furthermore, epigenetic modifications play a critical role in regulating macrophage polarization^[121,129,132]^. Processes such as DNA and RNA modification could be utilized to modulate or reinforce macrophage phenotypes. For instance, methyltransferase-like enzymes 3 (METTL3) and METTL14 have been shown to promote M1-like polarization through post-transcriptional methylation of STAT1 mRNA and myeloid differentiation primary response 88 (Myd88) mRNA^[129,131]^. Similarly, DNA methyltransferase-3β (DNMT-3b) facilitates pro-inflammatory polarization by methylating PPARγ^[131]^. In contrast, M2-like polarization was suppressed by employing histone deacetylase (HDAC) inhibitors, indicating that selectively enhancing certain HDAC activities may provide a targeted approach to modulating macrophage phenotypes^[124,131–132]^. This suggests a potential therapeutic strategy wherein mRNA delivery encoding epigenetic and posttranscriptional enzymes could be employed to enhance M2-like polarization in cardiac resident or infiltrating macrophages^[52,124,131]^.

Another potential target for modulating macrophage polarization lies within cytokine signaling networks^[117,121,130]^. Depending on the specific subtype, pro-regenerative polarization can be induced by the tissue niche^[121]^. For instance, M2a macrophages, which are strongly anti-inflammatory, involved in tissue repair, and are generated in response to IL-4 or IL-13 stimulation^[123]^. Similarly, M2c macrophages, which are also anti-inflammatory and contribute to tissue homeostasis, are stimulated by IL-10^[123]^. The feasibility of overexpressing key cytokines with mRNA has already been validated as a cancer immunotherapy in tumor models^[133–134]^. Building on this concept, delivering mRNA encoding specific cytokines offers a plausible therapeutic strategy to enhance pro-regenerative polarization within macrophages to repair the heart.

Comparable to shifting the phenotype of macrophages and recruited monocytes^[135]^, reinforcing regulatory phenotype in recruited CD4 T cells^[136]^ or blunting cytotoxicity in infiltrating CD8 T cells^[137]^ is known to improve myocardial recovery. Coding mRNA interventions offer substantial opportunities on this front by transiently overexpressing key transcription factors, signaling molecules such as catalytic-dead tyrosine-protein kinase (LCK), zeta-chain-associated protein kinase 70 (ZAP70), regulatory receptors CD5, dominant negative or signaling deficient molecules of modified LCK or ZAP70 or defective tissue homing receptors^[138]^. Nevertheless, caution is warranted with an active transcription factor acting across a wide swath of the T cell compartment as to not disrupt their many other important functions and self- and microbiome tolerance, alongside infectious disease elimination. Within CD4 T cells, exogenous FOXP3 is sufficient to induce a regulatory phenotype^[136]^ which can promote myocardial recovery after acute injury^[139]^. Importantly, CD4 T cells appear to maintain significant lineage plasticity, suggesting that the overall CD4 compartment may “reset” to baseline after the transient desired therapeutic effect. Naturally recruited lymphocytes like neutrophils, monocytes, and T cells could also serve as transient factories of anti-inflammatory cytokines such as IL-10^[140]^ or IL-13^[141]^.

Recent advances in CAR-T cell technology have been substantial^[142]^. CAR-T cells enable precise targeting of specific cell populations by recognizing a single cell surface antigen. This technology has demonstrated significant success in cancer therapy, particularly in B cell and plasma cell malignancies. Moreover, CAR-T cell technology is increasingly being applied to non-oncologic conditions, including autoimmune^[143–145]^ and CVDs^[143,146–148]^. As these innovative fields expand, we highlight only two pivotal studies that apply CAR-T therapy to myocardial diseases.

Various cardiovascular conditions, including acute injuries such as MI, chronic diseases like HF, and the effects of aging, contribute to the progression of cardiac fibrosis, ultimately impairing cardiac function^[149–150]^. This underscores the urgent need for the development of effective antifibrotic therapies, particularly those that directly remove pathogenic fibroblasts. Aghajanian *et al.*^[149]^ developed CAR-T cells that specifically eliminated activated fibroblasts, through targeting fibroblast activation protein (FAP), a cell surface marker exclusively expressed on activated fibroblasts. These FAP CAR-T cells demonstrated the ability to reduce cardiac fibrosis in a mouse model of cardiac pressure-overload injury induced by angiotensin II and phenylephrine (AngII/PE)^[149]^. It was demonstrated that in vivo induction of these CAR-T cells through the injection of targeted lipid nanoparticles encapsulating mRNA (tLNP/mRNA) encoding the FAP CAR is feasible^[6]^. These nanoparticles specifically targeted the surface marker CD5 on T cells, which is not required for T cell effector function^[6]^. This innovative approach offers distinct advantages. The traditionally labor-intensive, expensive, and complex process of ex vivo CAR-T cell generation can be significantly streamlined through mRNA delivery methods, offering rapid scalability and improved patient accessibility^[147–148]^. This approach also enhances safety by eliminating the need for lymphodepletion prior to CAR-T cell infusion, thereby reducing the risk of cytokine release syndrome, opportunistic infections, and secondary malignancies^[147]^. Additionally, conventional ex vivo-generated CAR-T cells permanently express CARs, with the potential to persist for decades in patients^[142]^. Although this permanence is beneficial for cancer therapies, it is undesirable in conditions such as fibrosis, which is an essential tissue maintenance response to severe injuries^[150]^. The transient nature of mRNA within cells allows for only temporary CAR-T cell activity^[6,151]^. This feature makes mRNA delivery an appealing strategy for developing safe CAR-T cells. However, critical questions remain regarding the local mechanisms of action of FAP CAR-T cells and tissue recovery pathways. Further research is necessary to evaluate potential adverse effects, including off-target activity, cardiac inflammation, and disruptions to essential tissue repair and remodeling processes due to fibroblast cell death^[146,152]^.

Another intriguing area of research lies in the study of cellular senescence, which is when cells lose proliferative capacity, permanently exit the cell cycle, and diminish functions^[153–154]^. The molecular mechanisms driving cellular senescence are not yet fully elucidated^[154]^. However, it appears to be associated with factors such as DNA damage, dysregulation of oncogenic proteins, increased production of ROS, and changes in metabolism to end up with a characteristic senescence-associated secretory phenotype (SASP)^[154–156]^. SASP is particularly noteworthy due to its role in promoting tissue dysfunction by secretion of pro-inflammatory and proteolytic molecules^[154–157]^. More comprehensive discussions of senescence and aging, particularly within the heart, have been reviewed by Chen *et al*.^[155]^ and Mehdizadeh *et al*.^[154]^.

The therapeutic targeting of cellular senescence is of significant interest and is currently being explored through various strategies, including modulation of the SASP, interference with pro-senescent stressors, and, notably, the elimination of senescent cells using senolytic therapies^[154]^. Alongside the proof-of-concept experiments with anti-fibrotic CAR-T cells, anti-senescent CAR-T cells recently emerged in the context of liver disease^[158]^. Amor *et al.*^[158]^ leveraged the cell surface protein urokinase plasminogen activator receptor (uPAR) which is predominantly expressed on senescent cells and in tissues with senescence-associated disorders^[157–158]^. The authors developed uPAR-targeting CAR-T cells, which were shown to effectively eliminate senescent cells in models of induced liver fibrosis^[158]^. This therapeutic approach holds significant potential for addressing cardiac aging and disease where senescence plays pathogenic roles such as MI, cardiac hypertrophy, cardiotoxic drug side effects, and fibrosis^[154–155,157]^. The integration of the previously discussed tLNP/mRNA delivery technique with cutting-edge anti-senescent CAR-T cell technology could offer a promising avenue for advancing targeted senolytic therapies including those aimed at the heart. Lastly, CD19 CAR-T cells developed against B cells in hematological cancers and, more recently, autoimmune diseases such as systemic lupus erythematosus^[143]^, may be beneficial in a subset of heart diseases where autoreactive IgG antibodies seem to contribute to cardiac pathology^[25]^.

An important avenue for future exploration lies in the expanding field of alternative CAR-based therapies^[147–148,159]^. Beyond T cells, CARs can be engineered into other immune cells, such as macrophages^[160–161]^ or Tregs^[136]^, broadening their therapeutic applications^[162]^. One potential usage of this alternative CAR technology is to direct macrophages’ phagocytic activity toward pathogenic fibrosis, should a suitable extracellular matrix antigen and CAR be identified. Additionally, CAR-macrophages have demonstrated the ability to target and degrade amyloid plaques in a mouse model of Alzheimer disease^[163]^. Pathogenic accumulation of amyloid proteins also drives certain CVDs^[164]^, suggesting that CAR-macrophages may also be beneficial to restore cardiac function and mitigate disease progression^[165]^. Importantly, targeted LNPs are being developed to deliver mRNA directly to monocytes in vivo^[166–167]^, supporting the translational potential of CAR-macrophage therapies into the cardiovascular clinic. Finally, Tregs expressing CAR molecules are being developed to calm inflammatory environments in certain autoimmune diseases including type 1 diabetes, systemic lupus erythematosus, and graft versus host disease^[136]^. This type of approach may be beneficial in acutely inflammatory diseases of the circulatory system such as myocarditis and ischemia-reperfusion injury.

Finally, major translational successes using RNA are on the immediate horizon in the form of gene editing within the liver. Given the transient nature of mRNA-expressed genome-editing enzymes, especially base editors, the risk of genotoxicity is significantly lowered compared to virally delivered endonucleases which can be re-activated. Furthermore, broad hepatic delivery by LNPs makes these permeant gene edits a reality from a single intravenous injection. Numerous therapies leveraging these technologies are in pre-clinical development and clinical trials, with several highlighted here that will have direct cardiovascular benefits. First, PCSK9 inactivation in the liver has been shown to permanently lower circulating levels of LDL cholesterol, a known risk factor for atherosclerosis and coronary heart disease^[45]^. Furthermore, the expression of TTR can be shut off by liver editing to limit the accumulation of amyloid plaques, protecting patients from further disease pathogenesis^[168]^. Using targeted delivery vehicles^[8]^, HSC editing could eventually mitigate CHIP or blood disorders like sickle cell anemia and β-thalassemia^[169]^. Finally, if delivery challenges are met, RNA-based editing to induce exon skipping may also dramatically improve the lives of patients with muscular dystrophy^[170]^. The rapidly expanding therapeutic field of mRNA-delivered base editors is already curing patients of disease^[171]^, including several with important cardiovascular implications.

## CONCLUSION

There are always significant challenges in translating potential therapies, especially when modulating pleiotropic molecules and cells. Many immune-related molecules can exert both protective and pathological effects depending on the context, timing, and disease state. For example, transforming growth factor beta (TGFβ) is both pro-inflammatory in tissues, especially as a potent activator of fibroblasts, yet largely anti-inflammatory in the context of T cells where it drives a regulatory phenotype^[11]^. Similarly, the systemic effect of IL-2 varies dramatically depending on dose^[172]^, which has historically posed significant clinical challenges. Innovative, carefully designed therapies which take timing, dose, and target specificity into account will inevitably revolutionize the treatment of CVDs. It is also critical to continue to investigate the fundamental molecular and cellular pathologies in each CVD, as successful therapies most often arise from thorough biological understanding of the disease. Furthermore, an otherwise perfect RNA-based therapy could be totally useless without efficient, safe delivery, or premature clearance from the body. Fortunately, significant efforts in academic and industrial laboratories have made significant strides toward developing high-quality delivery vehicles. As the number of these compounds continues to expand^[173]^, it is important to leverage the most appropriate chemistry for the intended therapeutic context. For example, certain LNPs trigger significant inflammation, whereas ideal for vaccines^[46]^ and anti-cancer therapeutics, this feature is undesirable when treating heart or autoimmune diseases. Finally, evaluating immune-modulating therapies in animal models presents significant challenges that should not be overlooked. The immune system is arguably the most evolutionarily divergent organ^[174–175]^; therefore, its multifaceted role in disease resolution is significantly more likely to be species-specific than other pathological processes. Consequently, immunomodulatory therapies may not exhibit the same regenerative or protective effects in rodent, canine, porcine, or non-human primate models due to fundamental differences in the underlying immune systems of each species.

Taken together, there are a seemingly infinite number of potentially powerful RNA-based therapies on the horizon that leverage potent mechanisms of immunocardiology. These advanced drugs will become important constituents of the burgeoning personalized medicine arsenal. As demonstrated by the widespread success of RNA COVID-19 vaccines, this powerful technology is both safe and scalable. These features will help to ensure that all patients have access to the advanced therapies they deserve.

## FUNDING

JGR is supported by the Swedish Cancer Foundation, Cancer Research Karolinska Institutet, and the Sjöberg Foundation. This work was supported by the Else-Kröner Excellence Fellowship of the EKFS (project number: 2023_EKES.04), a grant of the Deutsche Herzstiftung e.V. (German Heart Foundation), and research grants of the Deutsche Forschungsgemeinschaft (German Research Foundation, DFG; MU 4555/2-1, project number: 455425596; and MU 4555/5-1, project number: 546575044) to JM. MB is supported by the Swedish Research Council (2018-02330, 2020-06121, 2021-04779, 2021-01141, 2022-01313), the Knut and Alice Wallenberg Foundation (2021.0136, 2022.0021), the European Union’s Horizon Europe Framework Programme (ERC: 101041484 and Horizon Europe: 101057129), the Swedish Society for Medical Research (CG-22 0009), the Swedish Cancer Society (22 2237 Pj), the Karolinska Institutet, (2019-00969, 2021-00513, 2022-01719), the Center for Innovative Medicine (FoUI-988204), and the PolyBio Research Foundation (Balvi grant B43 and B90).

## AUTHOR CONTRIBUTIONS

JGR outlined, collected references, drafted, and revised the manuscript. CLP drafted portions of the manuscript. JM and MB supervised and provided critical revisions to the manuscript.

## CONFLICT OF INTEREST STATEMENT

JGR is listed on a patent concerning delivery of mRNA to T cells and has no financial competing interests. The other authors declare that they have no conflict of interest with regard to the content of this manuscript.

## DATA SHARING STATEMENT

Research data will be available to other researchers upon request to the corresponding author.
